# Supplementary material for: An Integrated Approach Reveals DNA Damage and Proteotoxic Stress as Main Effects of Proton Radiation in S. cerevisiae
Source: Int J Mol Sci. 2022 May 14;23(10):5493. doi: 10.3390/ijms23105493 (PMC9145671; doi:10.3390/ijms23105493)
Supplement: Supplementary file 1 [file ijms-23-05493-s001.zip › ijms-1711218-supplementary.pdf]

SUPPLEMENTARY MATERIAL

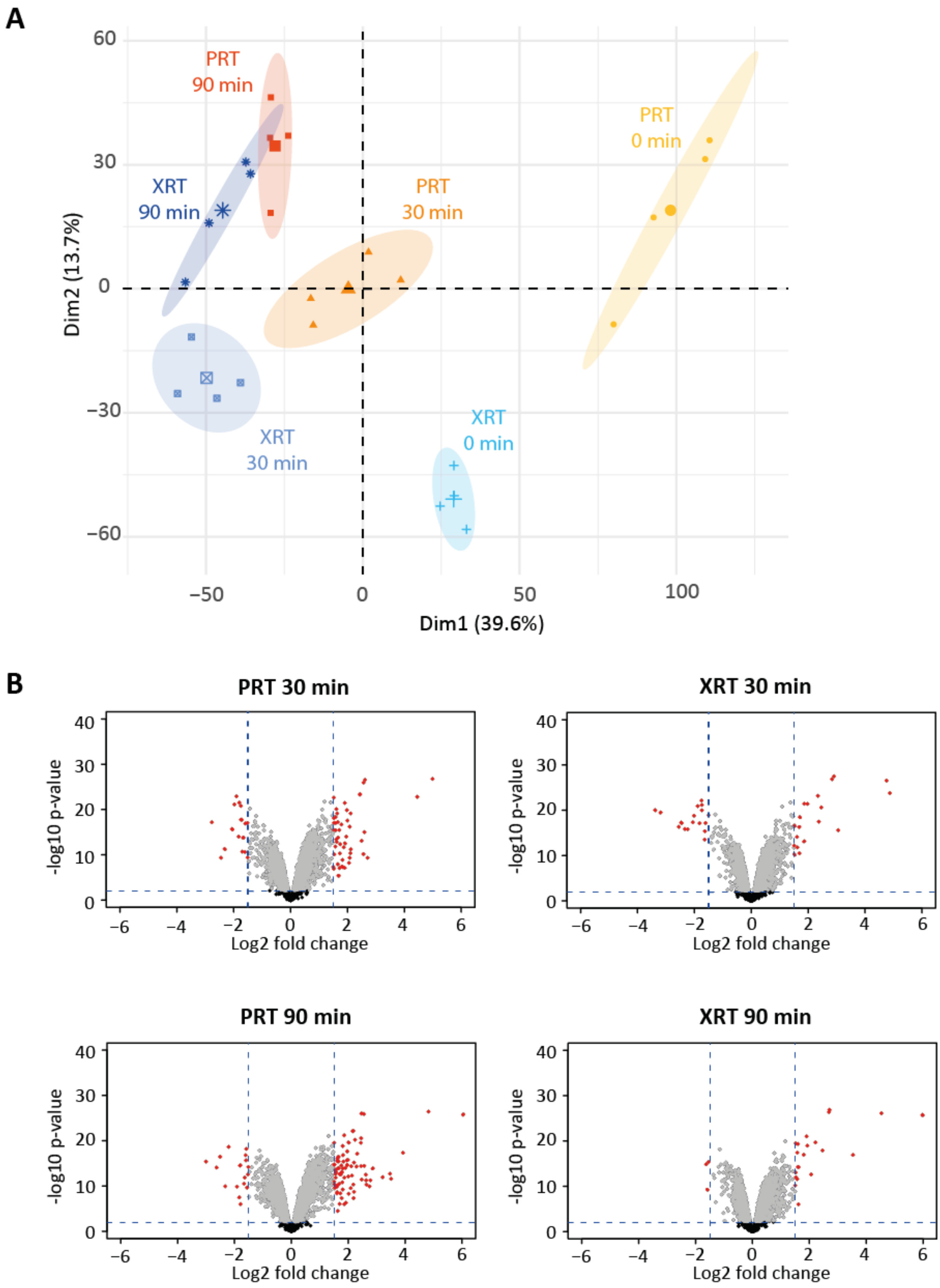

Supplementary Figure S1. Principal component analysis and Volcano plots for RNA-Seq data. **(A)** Principal Component Analysis (PCA) plot presenting all RNA-Seq samples, 4 replicates and 6 conditions. Replicates cluster together in this plot. Based on this plot, differences between irradiated and mock-irradiated conditions can be expected. Density ellipses per condition are drawn on the plot using the R package factoextra version 1.0.7 [1]. **(B)** Volcano plots are depicted using the log2FC and adjusted p-values generated in EdgeR. Dotted blue lines indicate the cut-off values for log2FC and FDR set at 1.5 and 0.01 respectively. Black dots represent genes for which neither of these cut-offs is met. Grey dots represent genes for which FDR < 0.01. Red dots represent genes for which both cut-offs are met.

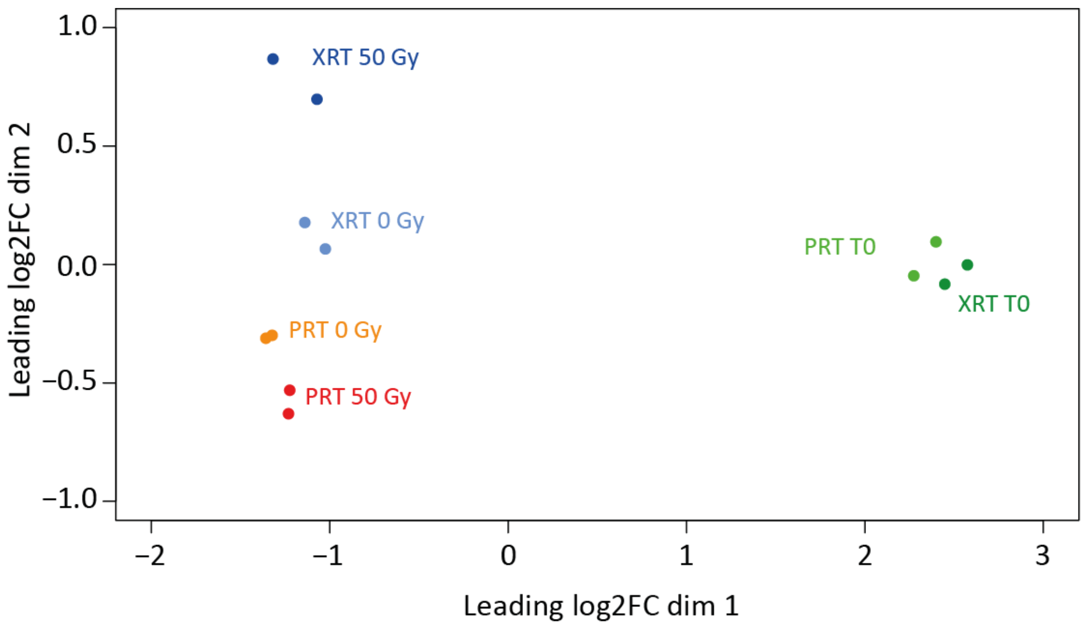

Supplementary Figure S2. Multidimensional scaling plot for Bar-Seq data. Multidimensional scaling (MDS) plot presenting all Bar-Seq samples in 2 replicates and 6 conditions. T0 timepoints (in green) represent the sample taken after pregrowing the pool and right before irradiation or mock irradiation. Other samples were grown for 8 generations after irradiation with 50 Gy protons or photons or mock irradiation.

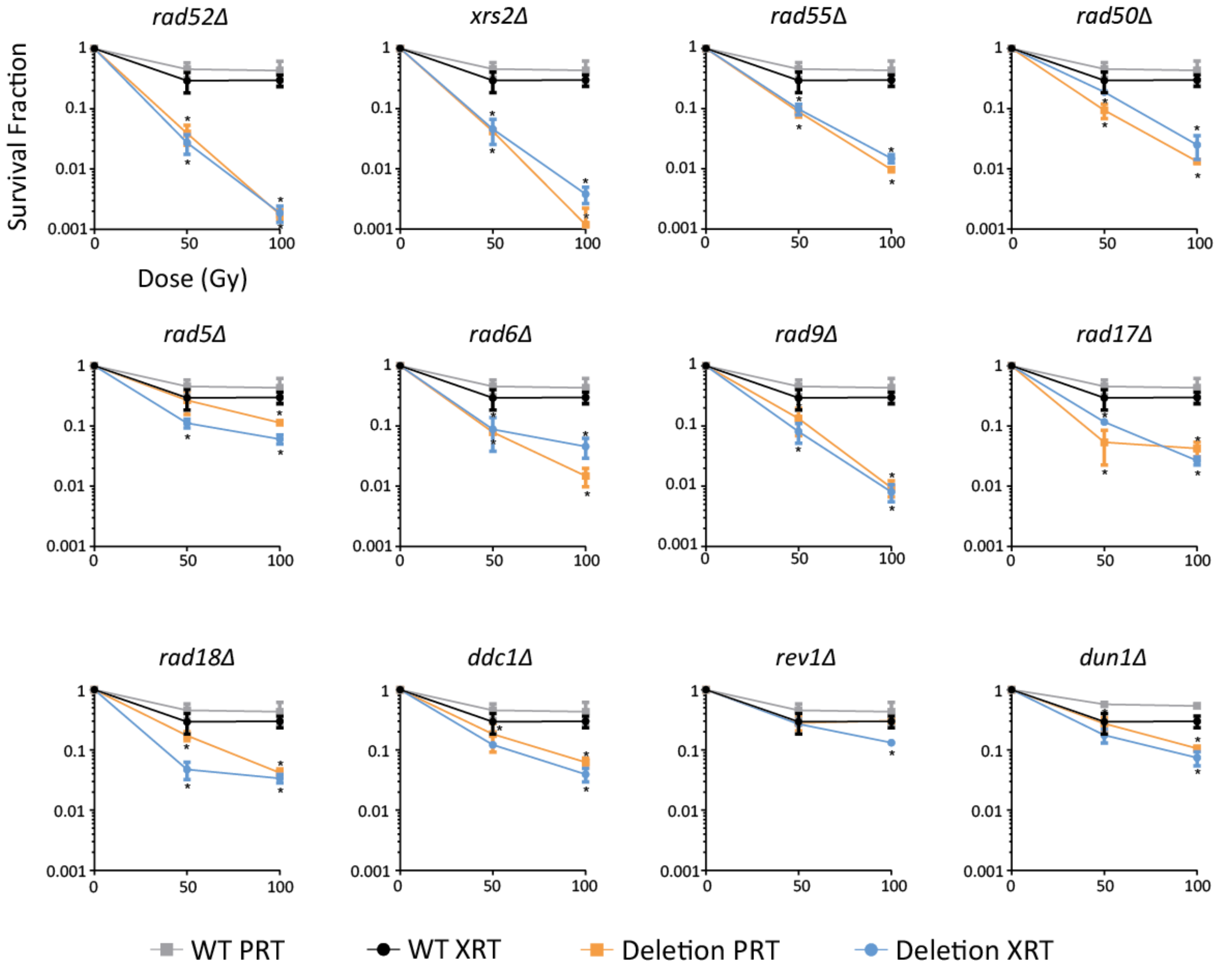

Supplementary Figure S3. Survival curves for sensitive deletion mutants involved in DNA repair. To confirm the results of the Bar-Seq experiment, the deletion mutants showing reduced survival were reconstructed by transformation (full genotypes are listed in Supplementary Table S1) and their sensitivity to both PRT and XRT was confirmed by plating assays. For 14 of the 43 genes, no deletion strain could be reconstructed after three attempts. For 12 of the 29 remaining candidate genes, all involved in DNA repair, the plating assays confirmed sensitivity to RT. Survival curves of the WT strain (KV447) are depicted for comparison and are used to perform statistics. P-values were calculated by multiple t-testing comparing the WT to the deletion per dose and within irradiation type. * $p < 0.05$.

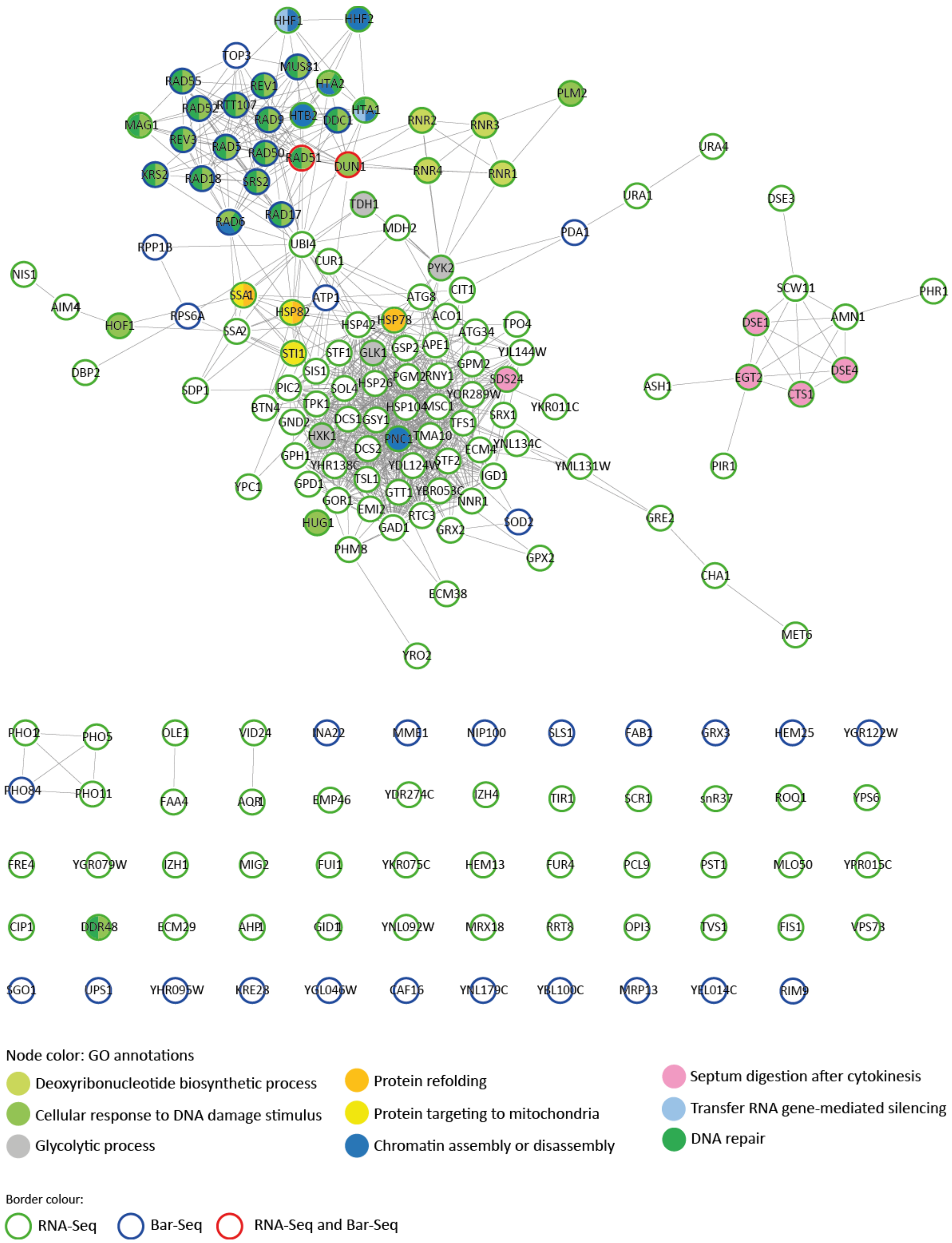

Supplementary Figure S4. Interaction network for genes found in RNA-Seq and Bar-Seq. Interaction network of genes found in the RNA-Seq and Bar-Seq using the previously defined cut-offs. The network was built in String and visualised in Cytoscape. Nodes with green are genes found only in the RNA-Seq experiment. Nodes with blue borders are genes only found in the Bar-Seq experiment. Nodes with red borders are genes found in both RNA-Seq and Bar-Seq. Node colour represents GO categories found by GSEA as performed in Figure 2.

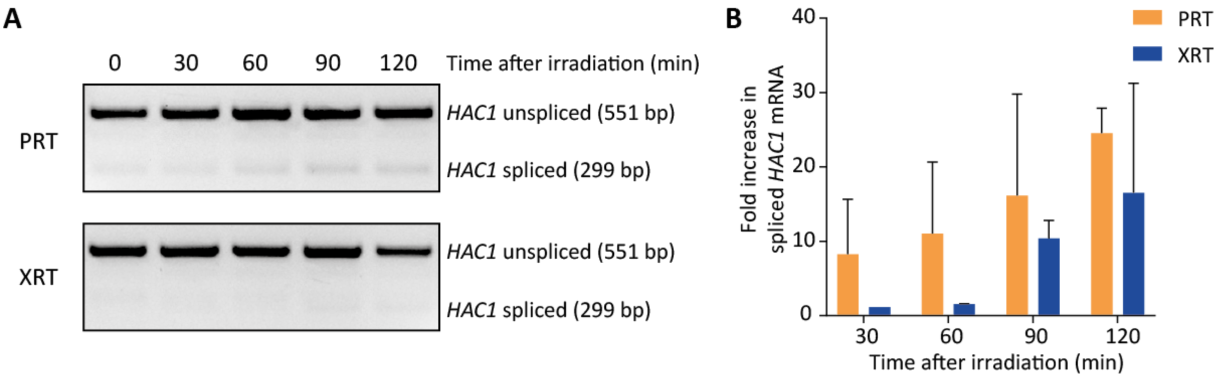

Supplementary Figure S5. Activation of the unfolded protein response is detected by determining *HAC1* mRNA splicing status. **(A)** Splicing status of *HAC1* mRNA was detected by RT-PCR across the intron. PCR products were visualised on a 2% agarose gel. **(B)** Quantification of gels in **(A)**. The amount of spliced and unspliced *HAC1* mRNA was quantified by densitometry in ImageJ. The ratio of spliced over unspliced *HAC1* mRNA was calculated and compared to the 0 min timepoint resulting in fold changes presented. Data are represented as the mean ± SEM for n = 3.

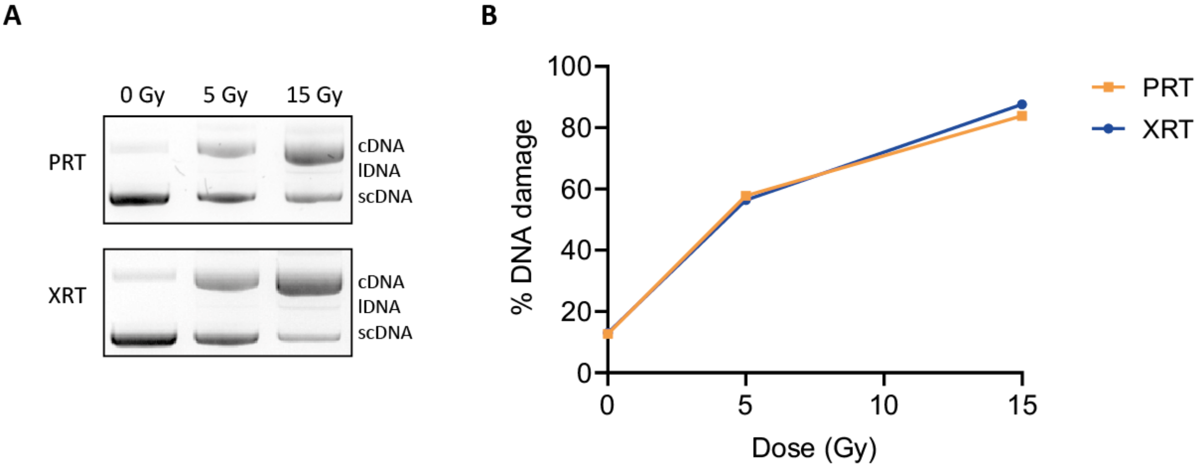

Supplementary Figure S6. Irradiation of φDNA reveals similar amounts of DNA damage after PRT and XRT. **(A)** Agarose gels of φDNA. 90 % supercoiled phiX174 RF1 DNA was mock-irradiated or irradiated with 5 and 15 Gy of protons and photons. Samples were run on a 1 % agarose gel to visualise the supercoiled and relaxed states. **(B)** Quantification of gels in **(A)**. The amount of DNA damage was quantified by densitometry in ImageJ. The percentage DNA damage was calculated as the ratio of cDNA + lDNA over the total amount of DNA loaded on the gel. Data are represented as the mean ± SEM for n = 3.

Supplementary Table S1. List of strains used in this study.

**Name**	**Genotype**	**Reference**
KV447	auxotrophic haploid S288c, BY4741 MATa *his3Δ1 leu2Δ0 met15Δ0 ura3Δ0*	[2]
LAV2	KV447 *rad52Δ::HYG*	This study
LAV3	KV447 *xrs2Δ::HYG*	This study
LAV4	KV447 *rad55Δ::HYG*	This study
LAV5	KV447 *rad50Δ::HYG*	This study
LAV8	KV447 *rad6Δ::HYG*	This study
LAV9	KV447 *rad17Δ::HYG*	This study
LAV12	KV447 *rad5Δ::HYG*	This study
LAV13	KV447 *rad9Δ::HYG*	This study
LAV15	KV447 *ddc1Δ::HYG*	This study
LAV22	KV447 *rad18Δ::HYG*	This study
LAV23	KV447 *rev1Δ::HYG*	This study
LAV74	KV447 *dun1Δ::HYG*	This study
LAV86	KV447 HSP104-YFP:KanMx	This study
LAV87	KV447 RAD52-YFP:KanMx	This study
LAV90	*pdr5Δ::HYG*	This study
LAV91	LAV86 *pdr5Δ::HYG*	This study

Supplementary Table S2. List of primers used in this study.

Primer Name	Sequence
LAV5_RAD52_KO_F	AAGAACTGCTGAAGGTTCTGGTGGCTTTGGTGTGTTGTTGCAGCTGAAGCTTCGTACGC
LAV6_RAD52_KO_R	AATGATGCAAATTTTTTATTTGTTTCGGCCAGGAAGCGTTCATAGGCCACTAGTGGATCTG
LAV7_RAD52_check_F	CTCTTCCCGTTAGTGATTCT
LAV8_RAD52_check_R	ATACGACACATGGAGGAAAG
LAV9_XRS2_KO_F	AGATGAGCAACAATACTGAGAAGGTGATAACTATAAATTTCAGCTGAAGCTTCGTACGC
LAV10_XRS2_KO_R	TATAATTTAATGAAATTGGAAATACTCGGAAAATTTATCACATAGGCCACTAGTGGATCTG
LAV11_XRS2_check_F	CGCCATCGTCATCACTAAAC
LAV12_XRS2_check_R	CGATCCGATACAGCTGAAAC
LAV13_RAD55_KO_F	ATAAGGAAAAGAACTTATTAATAATATATAATATGAAAATCAGCTGAAGCTTCGTACGC
LAV14_RAD55_KO_R	TTTTTTATTGTTTTCGGTTTTCGGTTTTTTTATTTTACTACATAGGCCACTAGTGGATCTG
LAV15_RAD55_check_F	CGTGTTCTCTTCTCATCGCG
LAV16_RAD55_check_R	AGCTGCATACCCCAATATGG
LAV17_RAD50_KO_F	CCATTGAGAGGCAAAAACAAGGGAACGACGGAAAGCAGGCCAGCTGAAGCTTCGTACGC
LAV18_RAD50_KO_R	CAATCAAAGTCTATCCCTTCGTAGATATTATGGGGTCTTTCATAGGCCACTAGTGGATCTG
LAV19_RAD50_check_F	TGCCTTTGTTCTTGCCTCAT
LAV20_RAD50_check_R	GCGTTATCACAAACTCGTCG
LAV29_RAD5_KO_F	AGGCCTTAGAAACACACCTAAAGTCTTACAGTATCACAATCAGCTGAAGCTTCGTACGC
LAV30_RAD5_KO_R	AATAATAAATAAAGTCTTTATATATGAGTATGTGGTATGACATAGGCCACTAGTGGATCTG
LAV31_RAD5_check_F	ATTCTGGACCTTTCTGCACG
LAV32_RAD5_check_R	TGACATTTCACAGAGGCAGC
LAV37_RAD6_KO_F	GATTATTTTTAGGCAGACAGAGACTAAAAGATAAAGCGTCCAGCTGAAGCTTCGTACGC
LAV38_RAD6_KO_R	ATCGGCTCGGCATTCATCATTAAGATTCTTTTGATTTTTCCATAGGCCACTAGTGGATCTG
LAV39_RAD6_check_F	ATGGTGACTACATTTCCCGG
LAV40_RAD6_check_R	GAAACTCACGATGAAGCCCT
LAV41_RAD9_KO_F	TAGAAAAGAGCATAGTGAGAAAATCTTCAACATCAGGGCTCAGCTGAAGCTTCGTACGC
LAV42_RAD9_KO_R	TCCCTTTCTATCAATTATGAGTTTATATATTTTTATAATTCATAGGCCACTAGTGGATCTG
LAV43_RAD9_check_F	TCTGATTAAACACGCGAGGA
LAV44_RAD9_check_R	CAACGTTGTTGGTTACCGTG
LAV45_RAD17_KO_F	CAGAACGGTGTGGAAACAAAGTAGTTGAAGGATTTCAACTCAGCTGAAGCTTCGTACGC
LAV46_RAD17_KO_R	TGAATGAAGTTCTGCGTTTTCTGCGATGCTGGATATTGACCATAGGCCACTAGTGGATCTG

LAV47_RAD17_check_F	ACTAAGGTGGCTAGAGACGC
LAV48_RAD17_check_R	AATGGGCTGGTTTGGGTAAG
LAV49_DDC1_KO_F	TAGTGTAACAATAACACAGCATAACTTTGCTTAGACATATCAGCTGA AGCTTCGTACGC
LAV50_DDC1_KO_R	TAATATTTACACGCCTTTATACTGATTTTGCATTATGGTTCATAGGCC ACTAGTGGATCTG
LAV51_DDC1_check_F	CGCGAATGAATCAAAGGCCG
LAV52_DDC1_check_R	TTTGTTCCCCGACCGTGATG
LAV85_RAD18_KO_F	GAGCATCACAGCTACTAAGAAAAGGCCATTTTTACTACTCCAGCTGA AGCTTCGTACGC
LAV86_RAD18_KO_R	TGCACAAGCTAACAAACAGGCCTGATTACATATACACACCCATAGGC CACTAGTGGATCTG
LAV87_RAD18_check_F	TACTTGCCCCGTTGCCTTGC
LAV100_REV1_KO_F	TCAAAATAAATCGATACTGCATTTCTAGGCATATCCAGCGCAGCTGA AGCTTCGTACGC
LAV101_REV1_KO_R	GCGTGTTTACTGTATGCTGAAATGTTTTTTTTTTTTAATCATAGGCCA CTAGTGGATCTG
LAV102_REV1_check_F	CGGCAACCTTTAAGCACCAA
LAV103_REV1_check_R	CTACTACAGGGAACACGGCA
LAV151_DUN1_KO_F	AGGGGCTTAACATACAGTAAAAAAGGCAATTATAGTGAAGCAGCTG AAGCTTCGTACGC
LAV152_DUN1_KO_R	CCAGATTCAAACAATGTTTTTGAAATAATGCTTCTCATGTCATAGGCC ACTAGTGGATCTG
LAV153_DUN1_check_F	TGACACTGCTCCAACTGGCC
LAV334_InternalHyg_R	TCGACAGACGTCGCGGTGAGTT
LAV335_HSP104_FLUO_F	GATAATGAGGACAGTATGGAAATTGATGATGACCTAGATATCGGTG ACGGTGCTGGTTTA
LAV336_HSP104_FLUO_R	ATTCTTGTTCGAAAGTTTTTAAAAATCACACTATATTAATCATCGATG AATTCGAGCTCG
LAV337_RAD52_FLUO_F	AGACCAAAGATCAATCCCCTGCATGCACGCAAGCCTACTATCGGTGA CGGTGCTGGTTTA
LAV338_RAD52_FLUO_R	ATGATGCAAATTTTTTATTTGTTTCGGCCAGGAAGCGTTTCATCGATG AATTCGAGCTCG
LAV339_HSP104_FLUO_check_F	CGAAGCTACTATAGGGGCTG
LAV340_RAD52_FLUO_check_F	CCGCAACAAAGATCGACACG
LAV341_InternalKanMx_R	GCACGTCAAGACTGTCAAGG
LAV342_HAC1_F	GATACGTTCACACCTTCACC
LAV343_HAC1_R	GAATTCAAACCTGACTGCGC
LAV346_ACT1_F	TTCCAGCCTTCTACGTTTCC
LAV347_ACT1_R	GAGCCAAAGCGGTGATTTCC
5_RE_PDR5_KO_Fw	CTTTTAAGTTTTCGTATCCGCTCGTTCGAAAGACTTTAGACAAAACAG CTGAAGCTTCGTACGC
6_RE_PDR5_KO_Rv	GTTTATTAAAAAAGGTCCATCTTGGTAAGTTTCTTTTCTTAACCAAAT TCAAAATTCTACATAGGCCACTAGTGGATCTG
13_RE_PDR5_KOcheck_Fw	GCACAGGATAAGTTGCAGGAAGC
14_RE_PDR5_KOcheck_Rv	CGTTCAGGGCCTGATGAGTG

Supplementary Table S3. List of plasmids used in this study.

Plasmid Name	Genotype	Reference
pCB1	loxP-TEF-HYG-TEF-loxP	[3]
pKT140	pFA6a-link-yECitrine-KanMx	[4]

References supplementary material

1. Kassambara, A.; Mundt, F. factoextra: Extract and Visualize the Results of Multivariate Data Analyses. R package version 1.0.7. **2017**.
2. Baker Brachmann, C.; Davies, A.; Cost, G.J.; Caputo, E.; Li, J.; Hieter, P.; Boeke, J.D. Designer Deletion Strains derived from Saccharomyces cerevisiae S288C: a Useful set of Strains and Plasmids for PCR-mediated Gene Disruption and Other Applications. *Yeast* **1998**, *14*, 115–132.
3. Brown, C.A.; Murray, A.W.; Verstrepen, K.J. Rapid Expansion and Functional Divergence of Subtelomeric Gene Families in Yeasts. *Curr. Biol.* **2010**, *20*, 895–903, doi:10.1016/j.cub.2010.04.027.
4. Sheff, M.A.; Thorn, K.S. Optimized cassettes for fluorescent protein tagging in Saccharomyces cerevisiae. *Yeast* **2004**, *21*, 661–670, doi:10.1002/yea.1130.
